# Spared Nerve Injury Increases the Expression of Microglia M1 Markers in the Prefrontal Cortex of Rats and Provokes Depression-Like Behaviors

**DOI:** 10.3389/fnins.2017.00209

**Published:** 2017-04-18

**Authors:** Ning Xu, Xiao-Hui Tang, Wei Pan, Ze-Min Xie, Guang-Fen Zhang, Mu-Huo Ji, Jian-Jun Yang, Mai-Tao Zhou, Zhi-Qiang Zhou

**Affiliations:** ^1^Department of Anesthesiology, Zhongda Hospital, School of Medicine, Southeast UniversityNanjing, China; ^2^Jiangsu Province Key Laboratory of Anesthesiology, College of Anesthesiology, Xuzhou Medical UniversityXuzhou, China; ^3^Department of Anesthesiology, Jinling Hospital, School of Medicine, Nanjing UniversityNanjing, China; ^4^Department of Anesthesiology, 101st Hospital of PLAWuxi, China

**Keywords:** neuropathic pain, depression, M1/M2 microglia, minocycline, inflammation

## Abstract

Pain and depression are frequently co-existent in clinical practice, yet the underlying mechanisms remain largely to be determined. Microglia activation and subsequent pro-inflammatory responses play a crucial role in the development of neuropathic pain and depression. The process of microglia polarization to the pro-inflammatory M1 or anti-inflammatory M2 phenotypes often occurs during neuroinflammation. However, it remains unclear whether M1/M2 microglia polarization is involved in the neuropathic pain induced by spared nerve injury (SNI). In the present study, the mechanical withdrawal threshold, forced swim test, sucrose preference test, and open field test were performed. The levels of microglia markers including ionized calcium-binding adaptor molecule 1 (Iba1), cluster of differentiation 11b (CD11b), M1 markers including CD68, inducible nitric oxide synthase (iNOS), interleukin-1β (IL-1β), IL-6, tumor necrosis factor-a (TNF-α), 8-hydroxy-2-deoxyguanosine (8-OH-dG), and M2 markers including CD206, arginase 1 (Arg1), IL-4 in the prefrontal cortex were determined on day 14 after SNI. The results showed that SNI produced mechanical allodynia and depressive-like behaviors, and also increased the expressions of microglia markers (Iba1, CD11b) and M1 markers (CD68, iNOS, IL-1β, TNF-α, and 8-OH-dG) in the prefrontal cortex. Notably, minocycline administration reversed these abnormalities. In addition, minocycline also promoted M2 microglia polarization as evidenced by up-regulation of CD206 and Arg1. In conclusion, data from our study suggest that SNI can lead to depression-like behaviors, while M1 polarization and consequent overproduction of pro-inflammatory cytokines plays a key role in the pathogenesis of neuropathic pain. The data furthermore indicate that modulation of inflammation by inhibition of M1 polarization could be a strategy for treatment of neuropathic pain, and might prevent the induction of neuropathic pain-induced depression symptoms.

## Introduction

Pain and depression are frequently co-existent. It is likely that pain can induce depression, while depression may enhance pain perception (Bair et al., 2003; Zhou et al., 2015). Neuropathic pain, one of the types of chronic pain, is a pain arising as a consequence of a primary lesion or disease affecting the somatosensory system (Treede et al., 2008). It is estimated that almost 50% of patients with major depression suffer from chronic pain (Maletic and Raison, 2009) and the prevalence of depression is around 30% in patients with neuropathic pain (Radat et al., 2013). The comorbidity of chronic pain and depression causes an increased disability, poor treatment response and consequently heavy disease burden (Dworkin and Gitlin, 1991; Arnow et al., 2006). Despite the broad spectrum of causes, little is known with regard to the mechanisms underlying the comorbid relationship, and consequently, few available treatments exist.

Neuroinflammatory activation is tended to be regarded as a causal role in the development of nerve injury-induced neuropathic pain and depression (Walker et al., 2014). Moreover, drugs or methods down-regulating neuroinflammation could alleviate the pain symptoms and depressive behaviors (Zhang et al., 2015; Luchting et al., 2016). Hence, modulation of neuroimmune response has been proposed for treatment of many central nervous system (CNS) disorders with an inflammatory component, including the comorbidity of neuropathic pain and depression (Kim et al., 2012; Walker et al., 2014; Bruning et al., 2015).

Microglia are resident macrophage-like immune cells in the CNS and play a critical role in both physiological and pathological conditions, including restoring the homeostasis of the CNS and driving the neuroinflammatory response of neurodegenerative disorders, respectively (Cherry et al., 2014). There are mainly two states of microglia polarization, “classical activation” (M1) and “alternative activation” (M2) (Colton, 2009; Colton and Wilcock, 2010). M1 microglia promote pro-inflammatory responses with excess tumor necrosis factor (TNF-α), interleukin-1β (IL-1β), inducible nitric oxide synthase (iNOS), and reactive oxygen species (ROS) production (Le et al., 2001; Block et al., 2007), contributing to neural network dysfunction. By contrast, M2 microglia are associated with up-regulation of anti-inflammatory cytokines and play a vital role in restoring homeostasis, such as dampening inflammation and wound healing (Ponomarev et al., 2007). Thus, modulation of microglia activation, including M1/M2 microglia polarization, for therapeutic purposes might be obtained by inhibiting the pro-inflammatory reaction and simultaneously promoting the anti-inflammatory effect. However, the status of microglia polarization in spared nerve injury (SNI)-induced neuropathic pain and depression-like behaviors has not been reported yet.

Based on these findings, we hypothesized that SNI causes an increase in M1 polarization and of inflammation markers in the prefrontal cortex, which may help to explain why patients with neuropathic pain frequently suffer from depression. In this study, we showed that SNI provoked depression-like behaviors and SNI-induced neuropathic pain was affected by microglia M1 polarization and overproduction of pro-inflammatory cytokines. Importantly, minocycline, a semisynthetic second-generation tetracycline with the anti-inflammatory properties, improved neuropathic pain and depression-like behaviors and attenuated M1 microglia polarization. In addition, it is well established that the prefrontal cortex plays an important role in the pathogenesis of pain (Metz et al., 2009), depression (Pan et al., 2014), and pain-related depression (Baliki et al., 2006; Guida et al., 2015), we therefore chose this brain region for biochemical analysis in the present study.

## Materials and methods

### Animal husbandry

**Figure 1:**
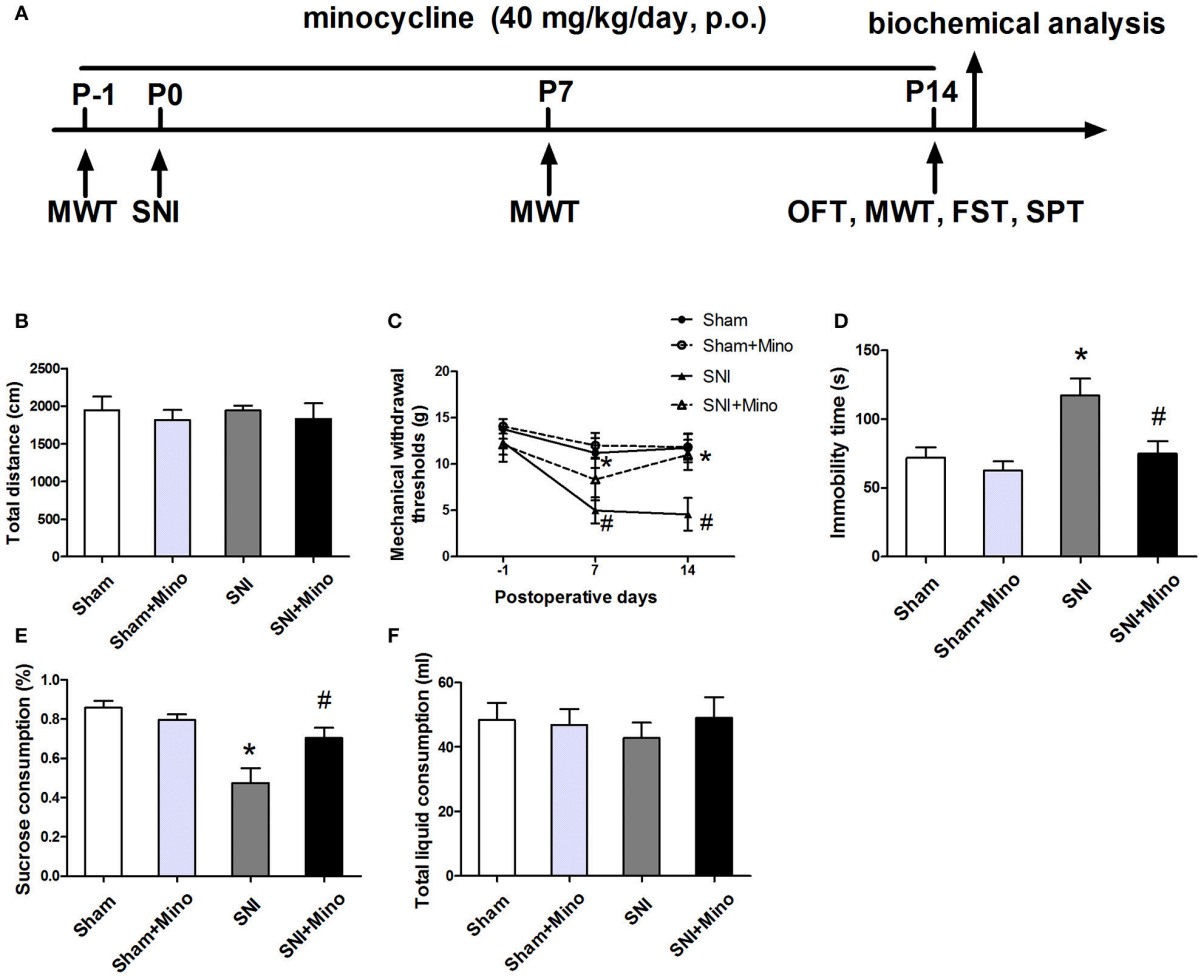
**Behavioral tests including mechanical allodynia and depressive-like behaviors were performed**. **(A)** Schematic timeline of the experimental procedure. The total distance **(B)** in the open field test was not significantly different among the four groups. **(C)** SNI elicited mechanical allodynia, while minocycline administration prevented SNI-induced decreases in mechanical withdrawal thresholds on days 7 and 14. **(D)** SNI increased the immobility time significantly in the forced swim test, while minocycline administration decreased the immobility time. **(E)** SNI reduced the sucrose consumption significantly and minocycline reversed this change. **(F)** No significant difference of the total liquid consumption was observed. The data are presented mean ± SEM (*n* = 12). ^*^P < 0.05 compared to the Sham group, ^#^*P* < 0.05 compared to the SNI group.

Male Sprague-Dawley rats (8 weeks old, 200–250 g) were purchased from the Animal Center of Jinling Hospital, Nanjing, China. Animals were grouped in 4–5 individuals per cage and were housed in in a standard condition (a 12-h light/dark cycle, room temperature of 22 ± 1°C and 50 ± 10% humidity, and food and water were available *ad libitum*) for 2 weeks before the start of tests. All procedures were approved by the Ethics Committee of Jinling Hospital, and were performed according to the Guideline for the Care and Use of Laboratory Animals from the National Institutes of Health. The flow chart of the study protocol was shown in Figure 1A.

### SNI surgery

SNI surgery was performed to induce neuropathic pain as previously described (Decosterd and Woolf, 2000). A rat was anesthetized by intraperitoneal (i.p.) injection of sodium pentobarbital (60 mg/kg) before surgery. The skin on the lateral surface of the right thigh of the rat was incised and the terminal branches of the sciatic nerve: the sural, common peroneal and tibial nerves were exposed by dissecting the biceps femoris muscle. The common peroneal and tibial nerves were tightly ligated with 4-0 silk at the point of trifurcation and then cut distal to the knot, followed by removing the distal nerve ends about 3–5 mm. The sural nerve was untouched during the surgery. For the sham-operated rats, the sciatic nerve was exposed in the same way but not ligated and cut.

### Experiment design and drug administration

The doses of minocycline from 20 to 50 mg/kg/day have been demonstrated as effective in attenuating microglia activation in the brain (Ekdahl et al., 2003; Raghavendra et al., 2003a). Thus, minocycline hydrochloride (Sangon Biotech, China) at a dose of 40 mg/kg/day was administered orally by rats' drinking water and minocycline solution was prepared at the concentration of 1 mg/mL in the drinking water every day (Hinwood et al., 2012). Minocycline or tap water was available *ad libitum* for rats via the drinking water except during behavioral tests, starting 24 h before sham/SNI surgery and consecutive 15 days[4 groups: Sham (*n* = 24), Sham + Minocycline (Mino) (n = 24), SNI (n = 24), and SNI + Mino (n = 24)]. For each group, half of them were for behavioral tests and the remaining half of them were for biochemical tests. Mechanical withdrawal thresholds (MWT) were assessed 1 day before SNI and on days 7 and 14 after SNI. The forced swim test (FST), sucrose preference test (SPT), and open field test (OFT) were performed on day 14 after SNI.

### Open field test

The open field test was carried out in a white opaque plastic chamber (100 × 100 × 40 cm, XR-XZ301; Shanghai Softmaze Information Technology Co., Ltd., Shanghai, China) as described in the previous study (Huang et al., 2015) with slight modification. Rats were gently placed in the center of the field, and the movement was recorded for 5 min with a video tracking system. Between subjects, 75% ethanol was used to clean the open field area.

### Von frey test

Mechanical allodynia was detected by the Von Frey test. The Von Frey monofilament test was performed as previously described (Wang et al., 2011). Rats were individually placed into plexiglass chambers over a mesh table and habituated for 15 min prior to the test. Von Frey filaments were used to stimulate the lateral 1/3 of right paws of animals starting with the 2.0 g filament and subsequently with logarithmically incremental stiffness from 0.6 to 15.0 g 1 day before the surgery or on days 7 and 14 after the surgery. Five trials for each paw were detected at 5-min intervals and the force (g) applied was recorded. Sudden paw withdrawal, flinching, and paw licking were defined as positive response. An up–down method was used, and 50% withdrawal threshold was calculated as described previously (Chaplan et al., 1994).

### Forced swim test

The forced swim test was performed as previously described with slight modification (Maeng et al., 2008). Rats were placed individually into Plexiglas cylinders (height, 65 cm; diameter, 30 cm) filled with 45 cm height of water, conditioned at 22–23°C. After 1-min habituation, the time when the rat kept floating passively or immobile in the water for a period of 5 min was recorded. After the test, subjects were dried with towel and then rest under a lamp for 30 min. The water was changed after each swim.

### Sucrose preference test

The sucrose preference test was performed as described by previous studies (Willner et al., 1987; Sigwalt et al., 2011). Animals were acclimated to the test room for at least 20 min before the test. Two bottles (1% sucrose solution vs. tap water) were presented to rats individually for 24 h. Each rat had free access to both 1% sucrose solution bottle and a tap water bottle and continued for 24 h. Sucrose and tap water intakes were measured by volume before and after the test. The total liquid consumption was the sum of sucrose water and tap water consumption. The sucrose preference of each rat was expressed as the percentage of sucrose water consumption divided by the total water consumption.

### Western blot

The prefrontal cortex were harvested after anesthetized with sodium pentobarbital (60 mg/kg, i.p.; Sigma, St Louise, MO, USA) on day 14 after surgery. The samples were placed on ice plate and homogenized in a lysis buffer containing 0.1% sodium deoxycholate, 1% Nonidet P-40, 0.1% SDS, 66 mM EDTA, 10 mMTris–HCl, (pH 7.4) with protease inhibitor cocktail. Homogenates were centrifuged at 4°Cat 12,000 rpm for 15 min and then the supernatants were collected. According to Bradford assay, the protein concentration was determined. Twenty micrograms of proteins were separated on 8–12% SDS-PAGE and transferred to polyvinylidene fluoride membranes (Millipore, Billerica, MA, USA), which was then blocked with 5% non-fat milk for 1 h at room temperature condition. Next the membranes were incubated with rabbit anti-CD11b (1:1,000; Abcam, Cambridge, UK), mouse anti-CD68 (1:1,000; Abcam, Cambridge, UK), rabbit anti-CD206 (1:500, proteintech, USA) and rabbit anti-β-tubulin (1:1,000; Bioworld, St. Louis Park, MN, USA) overnight at 4°C room. After washing in TBST (Tris Buffered Saline with Tween) for three times, the horseradish peroxidase-conjugated secondary antibodies (goat anti-rabbit and goat anti-mouse; Bioworld Technology, St. Louis Park, MN, USA) were diluted as 1:10,000 and the membranes were incubated for 1 h at room temperature. Chemiluminesence was used to detect the protein bands and Image J Software (Wayne Rasband, National Institute of Health, USA) was used to quantitate the band intensity.

### Immunofluorescence

Rats were anesthetized with 2% sodium pentobarbital in saline (60 mg/kg, i.p.; Sigma, St Louise, MO, USA) and perfused with saline and then perfused with 4% paraformaldehyde (PFA) in phosphate buffered saline (PBS) on day 14 after surgery. Brains were collected and fixed in the 4% PFA for 2 h and dehydrated in 30% sucrose at 4°C overnight. 10-mm-thick coronally sections of the prefrontal cortex were cut and pasted on glass slides. After blocking with 10% norm goat serum for 1 h at room temperature, the slices were incubated with primary antibodies: rabbit anti-CD11b (1:250; Abcam, Cambridge, UK), rabbit anti-Iba1 (1:500; Wako, Japan), mouse anti-CD206 (1:500; Biolegend, San Diego, USA), or mouse anti-8-hydroxy-20-deoxyguanosine (8-OH-dG; 1:200; Santa Cruz Biotechnology, Dallas, TX, USA) in 1% BSA at 4°C overnight. After washing with PBS for 3 × 5 min, the slices were exposed to the secondary antibodies Alexa fluor 488/549 goat anti-rabbit and Alexa fluor 488/549 goat anti-mouse (1:400; Bioworld Technology, St. Louis Park, MN, USA), and DAPI (1:1,000; Sigma, St. Louis, MO, USA) for 1 h at room temperature. A confocal microscope (Leica, TCS SP2, Germany) was used to capture the fluorescent images. The immunofluorescence intensity was calculated by Image J (Wayne Rasband, National Institute of Health, USA).

### Quantitative real-time PCR (qRT-PCR)

Total RNA was extracted from the prefrontal cortex and then isolated with Trizol (Takara Bio Inc). HiScriptQ RT SuperMix (Vazyme, Nanjing, China) was used to synthesize cDNA. qRT-PCR was performed by the ABI StepOne™ Real-Time PCR System (Applied Biosystems, Foster City, CA, USA) with SYBR Green PCR Master Mix (Vazyme, Nanjing, China) and analyzed using StepOne Software v2.3 (Applied Biosystems). The sequences of primers used for qRT-PCR amplification are available in Table 1. The cycling conditions were 95°C for 5 min and 40 cycles of 95°C for 10 s followed by 60°C for 30 s. GAPDH was used to normalize gene expression data and relative gene expression levels were calculated by ΔΔCT method.

**Table 1:** **The primer sequences using qRT-PCR of microglia markers and the reference gene**.

**Microglia markers (5′–3′)**			
CD11b	GAGTGTGATCCAGCTTGGTG	Arginase 1	TCCGCTGACAACCAGCTCTG
	GGACATATTCACAGCCTCTG		GTCCACATCTCGCAAGCCGA
CD68	CCTTACGGACAGCTTACCTT	CD206	CTGAACTGGCTACCAGGAAG
	CCAGGTGAATTGCTGGAGAA		CAGGCAGTAGGCACATCACT
IL-1β	TTGCTTCCAAGCCCTTGACT	IL-4	CCTTGCTGTCACCCTGTTCTG
	CTCCACGGGCAAGACATAGG		TGCATGGAGTCCCTTTTTCTG
TNF-α	CATGAGCACGGAAAGCATGA	GAPDH	TGCCACTCAGAAGACTGTGG
	CCACGAGCAGGAATGAGAAGA		TTCAGCTCTGGGATGACCTT
iNOS	CCTGGTGCAAGGGATCTTGG		
	GAGGGCTTGCCTGAGTGAGC		

### Enzyme-linked immunosorbent assay (ELISA)

Pro-inflammatory cytokines of TNF-α, IL-1β, and IL-6 and anti-inflammatory cytokine of IL-4 in the prefrontal cortex were quantified by the ELISA. Rats were sacrificed to obtain the prefrontal cortex on day 14 after surgery. The tissue samples were weighed and homogenized. Homogenates were centrifuged at 4°C for 10 min at 2,500 rpm and then collected the supernatants. Bradford method was used to quantify the protein concentration. The protocol was provided by the manufacturer (R&D Systems, Minneapolis, MN, USA) and the concentration of these cytokines was calculated by the standard curves, which was performed with the Optical Density (OD) tested at 450 nm.

### Statistical analysis

All analyses were performed by SPSS 17.0 software (version 17.0, IL, USA) and data are presented as mean ± SEM. Comparisons were assessed by one-way ANOVA followed by a Tukey test. Mechanical allodynia was analyzed by repeated measures two-way ANOVA followed by a Bonferroni test. Significant statistical difference was regarded as *P* < 0.05.

## Results

### SNI induced mechanical allodynia and depressive-like behaviors, which were attenuated by chronic minocycline administration

To evaluate the locomotor activity of rats, the open field test was performed. There was no significant difference in the total distance among the 4 groups (Figure 1B), suggesting neither SNI nor minocycline administration influenced the locomotor activity. Mechanical allodynia and depressive-like behaviors were used to verify the occurrence of neuropathic pain and depression, respectively. On day 7 or 14 after surgery, SNI induced increased mechanical withdrawal thresholds, which was reversed by chronic minocycline administration (Figure 1C, *P* < 0.05). The forced swim test and sucrose preference test were used to assess the depressive-like behaviors. The immobility time was longer and the sucrose consumption was less in the SNI rats than the sham-operated rats on day 14 after surgery, which were reversed by minocycline administration (Figures 1D,E, *P* < 0.05). There was no significant difference in the total liquid consumption among the 4 groups (Figure 1F). Moreover, there was no difference between the Sham group and the Sham + Mino group in the behavioral tests.

### SNI induced microglia activation in the prefrontal cortex, which was attenuated by chronic minocycline administration

**Figure 2:**
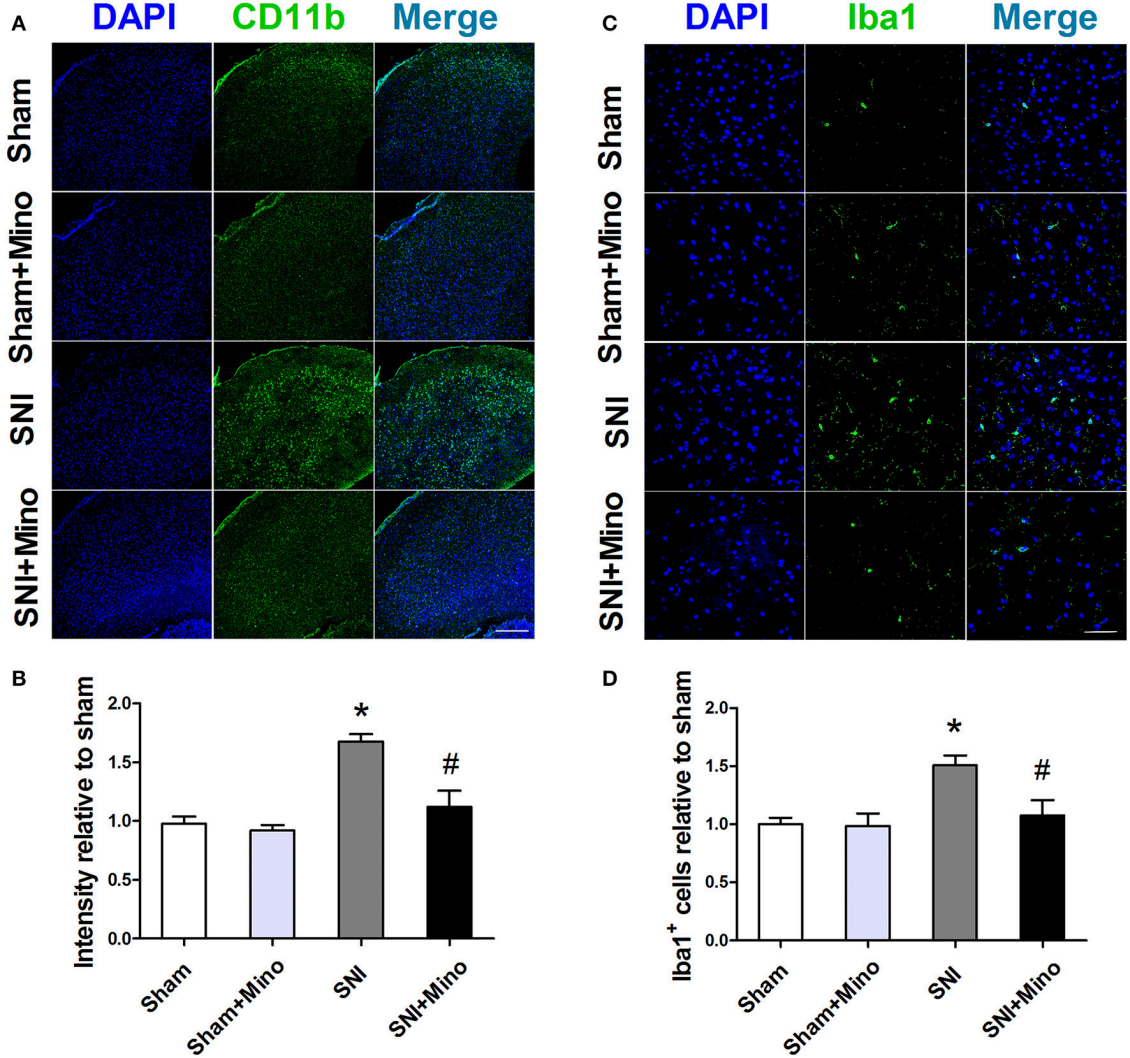
**Immunofluorescence staining to detect microglial activation in the prefrontal cortex. (A)** Representative images of CD11b (green) in the prefrontal cortex. **(B)** Minocycline significantly attenuated SNI-induced fluorescence intensity increase of CD11b in the prefrontal cortex. (Scale bar = 300 μm) **(C)** Representative images of Iba1 (green) in the prefrontal cortex. **(D)** Minocycline significantly attenuated SNI-induced fluorescence intensity increase of Iba1 in the prefrontal cortex. (Scale bar = 50 μm). The data are expressed as the mean ± SEM of 4 rats in each group. ^*^P < 0.05 compared to the Sham group, ^#^*P* < 0.05 compared to the SNI group. DAPI (4′,6-diamidino-2-phenylindole) staining is shown in blue.

To evaluate whether SNI could induce microglia activation in the prefrontal cortex, microglia markers including CD11b and Iba-1 were detected by immunofluorescence. SNI up-regulated the expression of CD11b (Figures 2A,B, *P* < 0.05) and the number of Iba1-positive cells (Figures 2C,D, *P* < 0.05) in the prefrontal cortex, whereas minocycline administration alleviated these abnormalities (Figure 2). There was no difference between the Sham group and the Sham + Mino group in the expression of CD11b and Iba1.

### SNI induced different changes in M1/M2 microglia markers in the prefrontal cortex, which were altered by chronic minocycline administration

**Figure 3:**
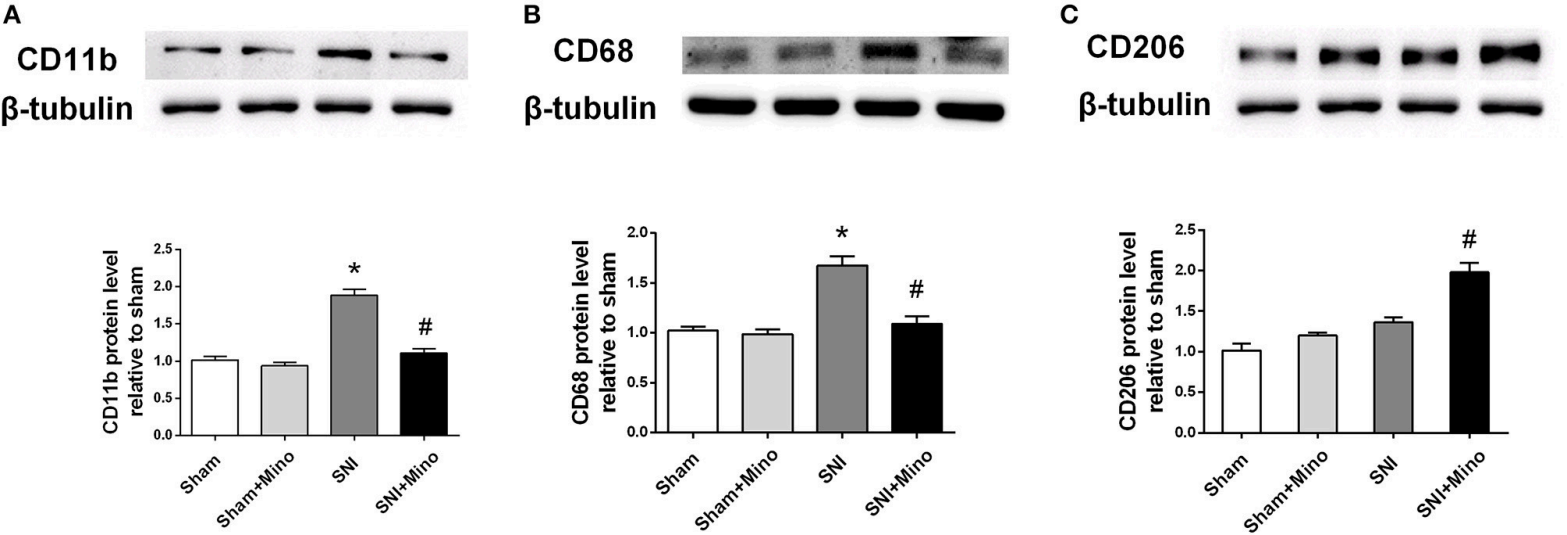
**Western blot results of the prefrontal cortex. (A)** A representative blot and quantitative analysis of CD11b in the prefrontal cortex. **(B)** A representative blot and quantitative analysis of CD68 in the prefrontal cortex. **(C)** A representative blot and quantitative analysis of CD206 in the prefrontal cortex. β-tubulin was included as control. The data are expressed as the mean ± SEM of 4 rats in each group. ^*^P < 0.05 compared to the sham group, ^#^*P* < 0.05 compared to the SNI group.

To quantify microglial M1 and M2 polarization at the protein level, the expressions of CD11b (a microglia marker), CD68 (a M1 microglia marker) and CD206 (a M2 microglia maker) were measured by the Western blot. SNI increased the expressions of CD11b and CD68 but not CD206 in the prefrontal cortex (Figure 3, *P* < 0.05), which was blocked by minocycline administration. In addition, minocycline administration increased the expression of CD206 after SNI in the prefrontal cortex (Figure 3, *P* < 0.05). There was no difference between the Sham group and the Sham + Mino group in the expression of CD11b, CD68 and CD206.

### Chronic minocycline administration reduced SNI-induced microglia activation and elicited up-regulation of M2 microglia in the prefrontal cortex

**Figure 4:**
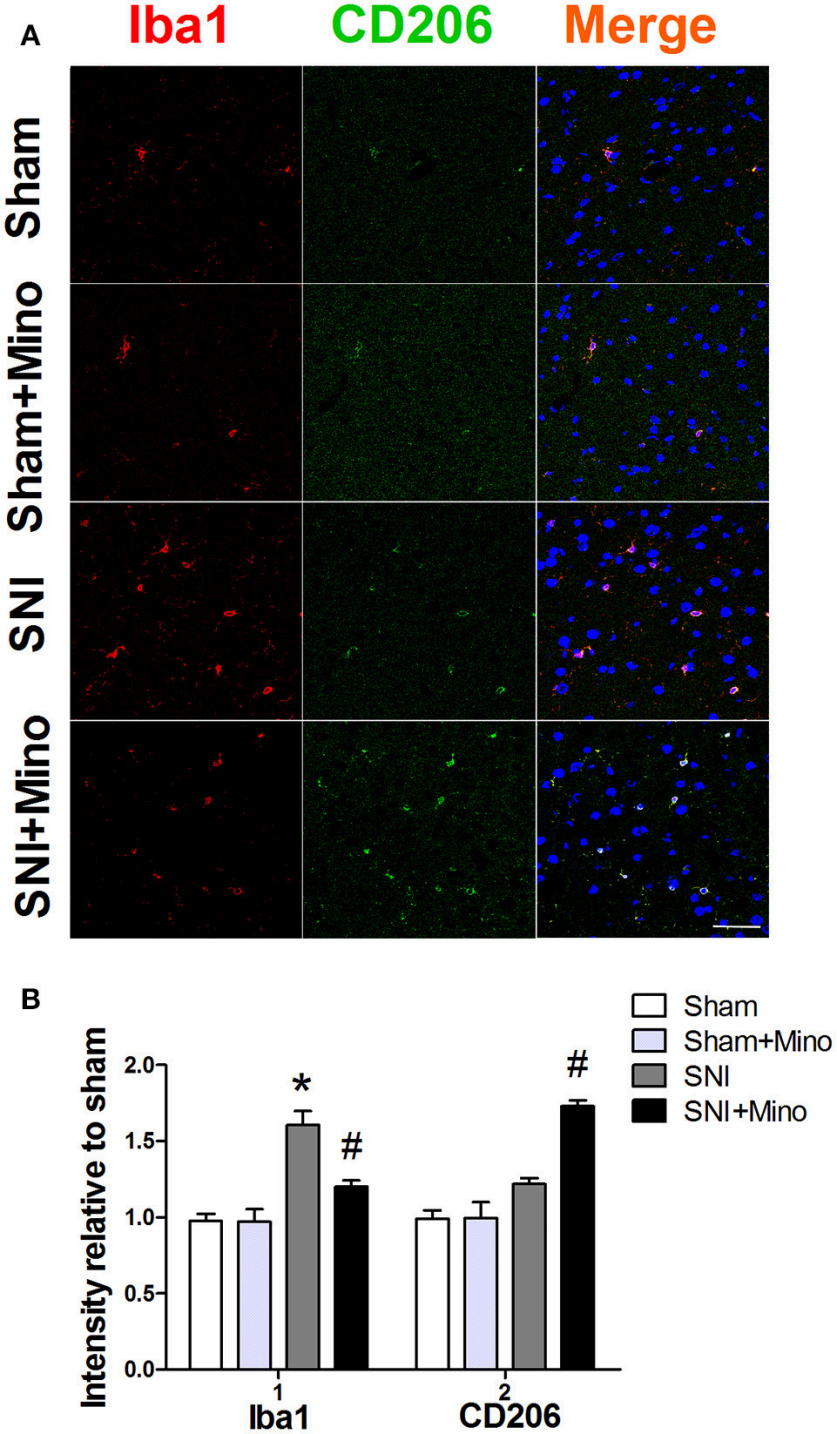
**Double-immunofluorescence staining to detect co-localization of Iba1 and CD206 in the prefrontal cortex on day 14 post-SNI. (A)** Representative images of Iba1 (red) and CD206 (green) in the prefrontal cortex. (Scale bar = 50 μm). **(B)** Quantification of Iba1 and CD206 fluorescence in the prefrontal cortex. The data are expressed as the mean ± SEM of 4 rats in each group. ^*^P < 0.05 compared to the Sham group, ^#^*P* < 0.05 compared to the SNI group. DAPI staining is shown in blue.

Since the results of Western blot have shown the similar trend between microglia marker CD11b and M1 microglia marker CD68 but not M2 microglia marker CD206, we used double-immunofluorescence staining to detect the co-localization of M2 microglia marker CD206 and another microglia marker Iba1 in the prefrontal cortex thus to further observe the trend of M2 microglia on day 14 after SNI. The co-localization result showed SNI increased the expression of Iba1 in the prefrontal cortex (Figure 4, *P* < 0.05), but did not affect the expression of CD206. However, minocycline administration blocked the change of Iba1 (Figure 4, *P* < 0.05) and increased the expression of CD206 (Figure 4, *P* < 0.05). There was no difference between the Sham group and the Sham + Mino group in the expression of Iba1 and CD206.

### SNI alters the ratio between M1 and M2 polarization

**Figure 5:**
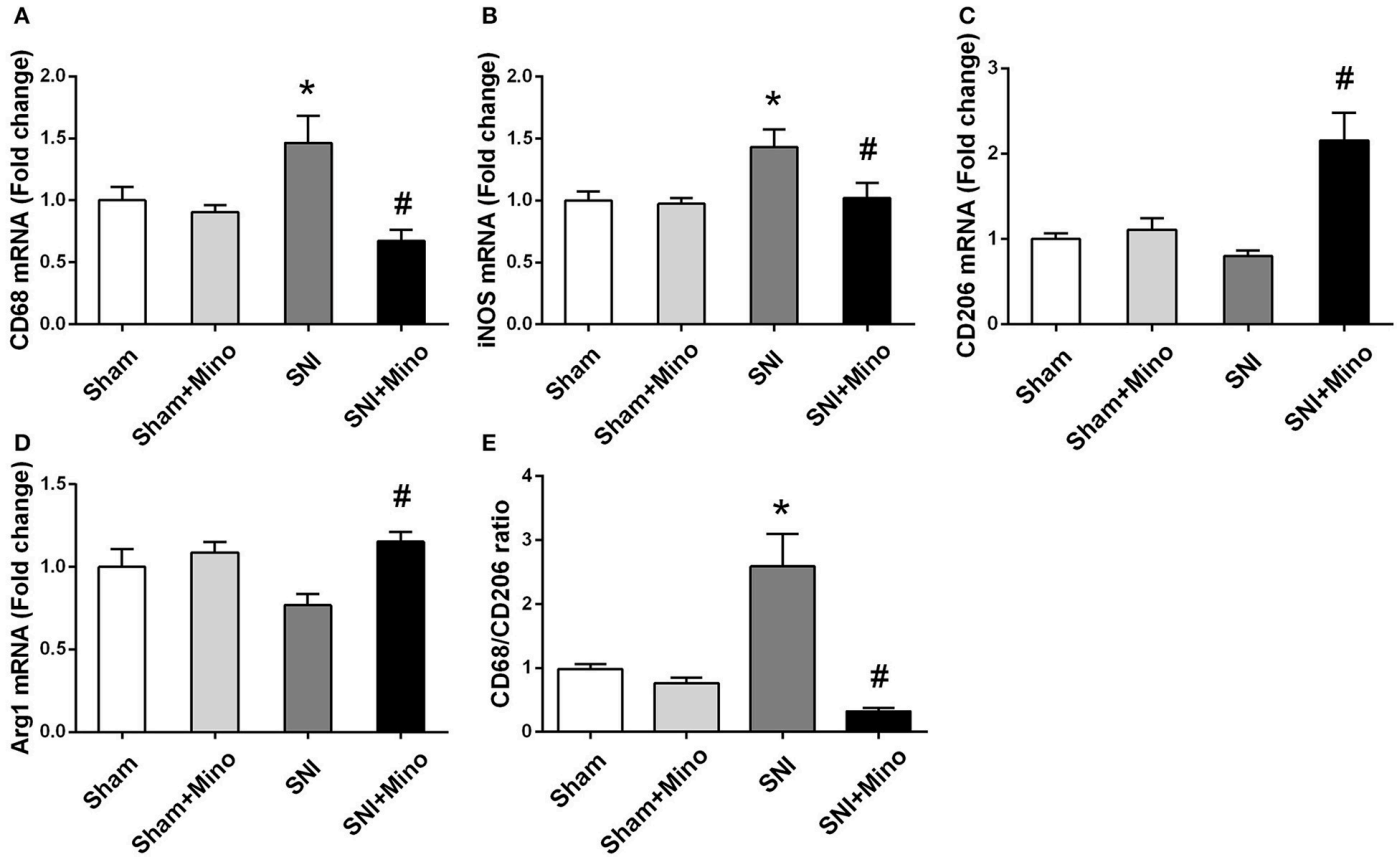
**mRNA expressions of M1/M2 microglia markers in the prefrontal cortex**. Fold increase of M1 markers (CD68, iNOS) **(A,B)**; Fold increase of M2 microglia markers (Arg1, CD206) **(C,D)**. Fold increase of the ratio of CD68/CD206 **(E)**. The data are expressed as mean ± SEM; *n* = 4 for each group; ^*^P < 0.05 compared to the Sham group, ^#^*P* < 0.05 compared to the SNI group.

To evaluate the alteration of M1/M2 microglia polarization at the genetic level, M1/M2 microglia markers were detected by qRT-PCR. SNI induced the increase of CD68 and iNOS (M1 markers; Figures 5A,B, *P* < 0.05) but did not affect CD206 and Arg1 (M2 markers; Figures 5C,D). However, minocycline administration rescued the increase of M1 microglia markers and increased M2 microglia markers (Figures 5A–D, *P* < 0.05). In addition, CD68 (M1 microglia marker)/CD206 (M2 microglia marker) ratio (Figure 5E, *P* < 0.05) was increased following SNI, whereas minocycline administration reversed the abnormalities, suggesting that minocycline redirected M1 activation to M2 activation. There was no difference between the Sham group and the Sham + Mino group in the expression of CD68, iNOS, CD206, Arg1, and CD68/CD206 ratio.

### SNI alters cytokine expression in the prefrontal cortex

**Figure 6:**
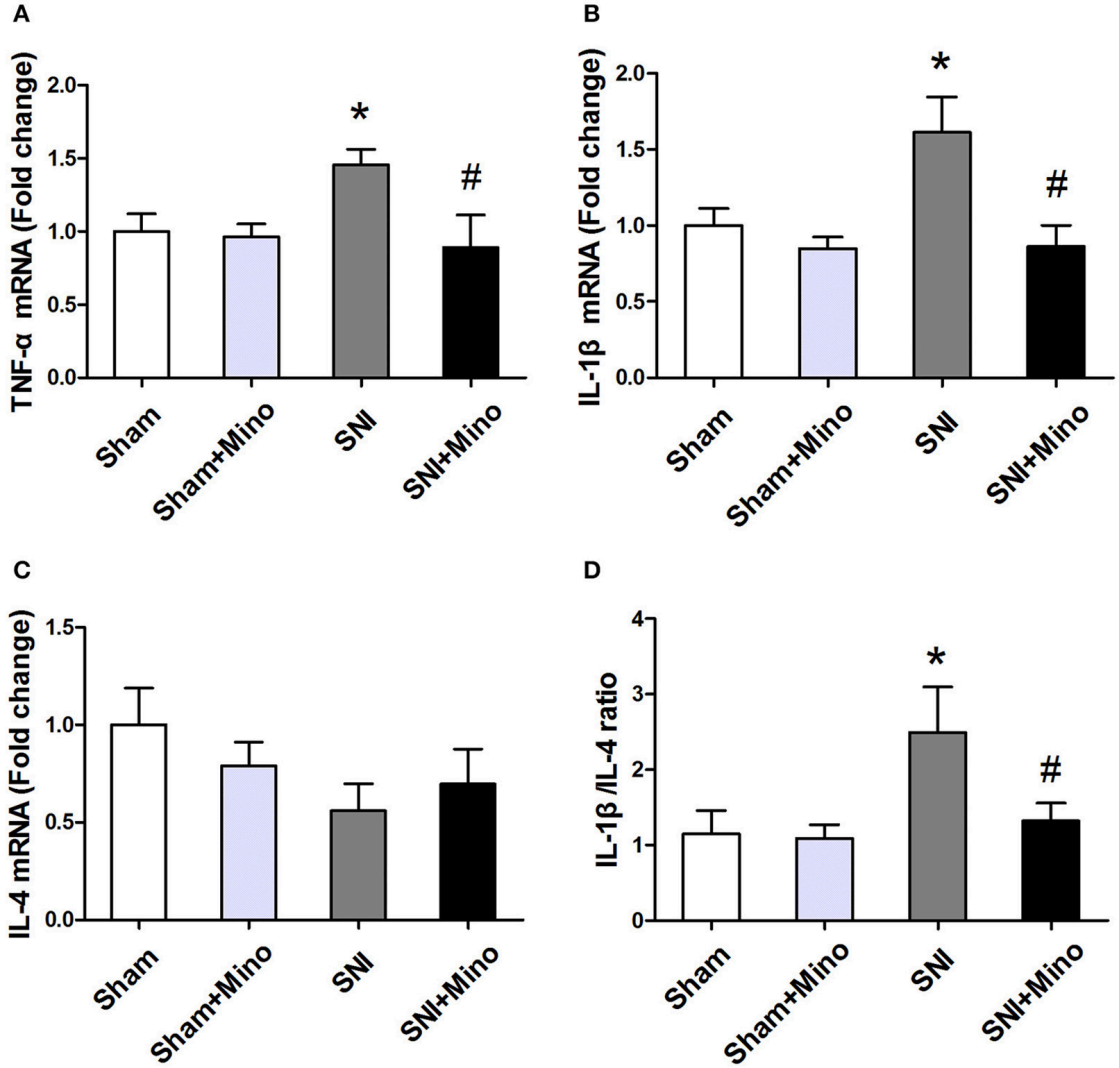
**mRNA expressions of different inflammatory cytokines in the prefrontal cortex**. Fold increase of pro-inflammatory cytokines (TNF-α, IL-1β) **(A,B)**; Fold increase of M2 anti-inflammatory cytokine (IL-4) **(C)**. Fold increase of the ratio of IL-1β/IL-4 **(D)**. The data are expressed as mean ± SEM; *n* = 4 for each group; ^*^P < 0.05 compared to the Sham group, ^#^*P* < 0.05 compared to the SNI group.

To evaluate the changes of inflammatory cytokines at mRNA level, qRT-PCR was performed. SNI increased the mRNA level of pro-inflammatory cytokines (TNF-α and IL-1β; Figures 6A,B, *P* < 0.05) but did not affect the mRNA level of anti-inflammatory cytokine (IL-4; Figure 6C) in the prefrontal cortex. Minocycline administration reduced the mRNA level of pro-inflammatory cytokines (Figures 6A,B, *P* < 0.05) but failed to increase the mRNA level of anti-inflammatory cytokine (Figure 6C). In addition, IL-1β/IL-4 ratio was increased following SNI, whereas minocycline administration reversed this abnormity (Figure 6D, *P* < 0.05), suggesting that minocycline redirected pro-inflammatory state to anti-inflammatory state. There was no difference between the Sham group and the Sham + Mino group in the expression of TNF-α, IL-1β, IL-4, and IL-1β/IL-4 ratio.

### SNI induced changes of pro- and anti-inflammatory cytokines in the prefrontal cortex, which were affected by chronic minocycline administration

**Figure 7:**
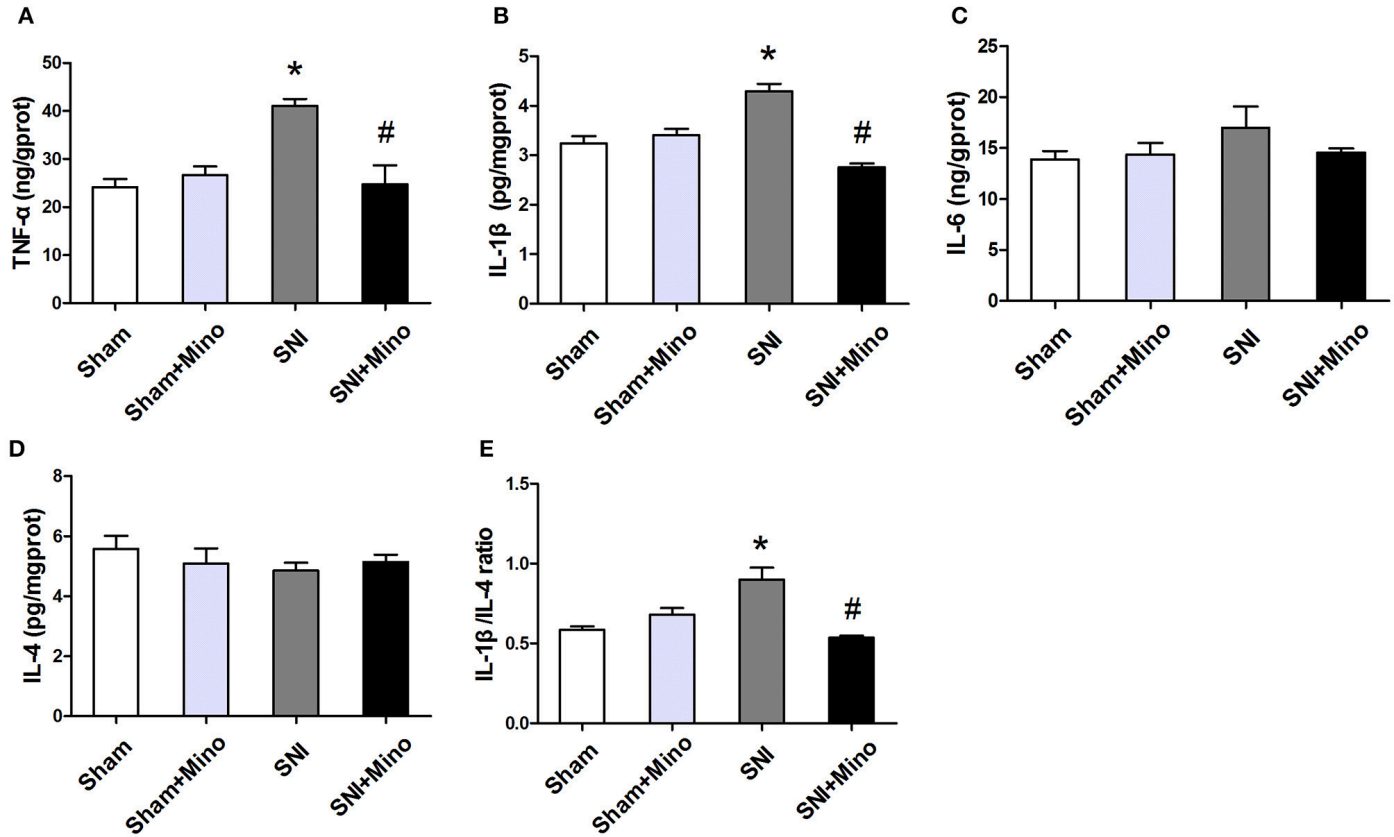
**ELISA analysis of different inflammatory cytokines in the prefrontal cortex. (A,B)** SNI-induced increase in pro-inflammatory cytokines TNF-α and IL-1 β were alleviated by minocycline. **(C)** No significantly difference of IL-6 was found among the 4 groups. **(D)** There was no difference of IL-4 among the 4 groups. **(E)** SNI increased the ratio of IL-1β/IL-4 and minocycline decreased the ratio. The data are expressed as the mean ± SEM of 4 rats in each group. ^*^P < 0.05 compared to the Sham group, ^#^*P* < 0.05 compared to the SNI group.

To measure the levels of pro-inflammatory cytokines (TNF-α, IL-1β, and IL-6) and anti-inflammatory cytokine (IL-4) in the prefrontal cortex, ELISA was applied. The levels of IL-1β and TNF-α were increased in the prefrontal cortex, whereas minocycline administration reversed the SNI-induced overproduction of TNF-α and IL-1β (Figures 7A,B, *P* < 0.05). In addition, there was no significant difference in IL-6 and IL-4 levels among the 4 groups (Figures 7C,D). Similarly, IL-1β/IL-4 ratio was increased following SNI, whereas minocycline administration reversed this change (Figure 7E, *P* < 0.05). There was no difference between the Sham group and the Sham + Mino group in the expression of TNF-α, IL-1β, IL-6, IL-4, and IL-1β/IL-4 ratio (Figure 7).

### SNI induced over-production of ROS in the brain, which was alleviated by minocycline

**Figure 8:**
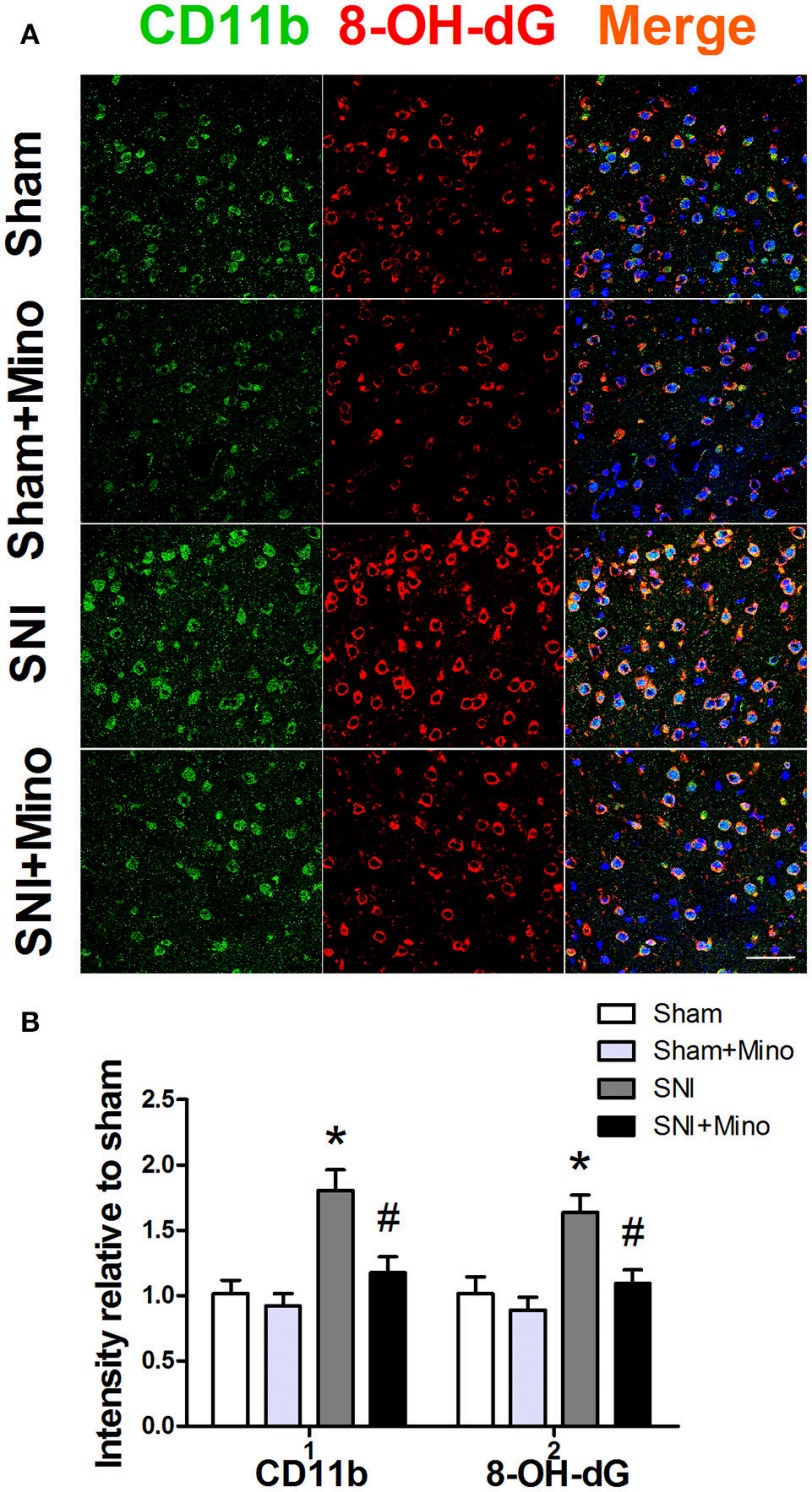
**Immunofluorescence staining to detect ROS in the prefrontal cortex. (A)** Representative images of CD11b (green) and 8-OH-dG (red) in the prefrontal cortex. **(B)** SNI significantly increased fluorescence intensity of CD11b and 8-OH-dG in the prefrontal cortex, which was attenuated by minocycline. (Scale bar = 50 μm). The data are expressed as the mean ± SEM of 4 rats in each group. ^*^P < 0.05 compared to the Sham group, ^#^*P* < 0.05 compared to the SNI group. DAPI staining is shown in blue.

Since M1 microglia activation produces ROS apart from iNOS, we finally determined whether SNI could lead to augmented oxidative stress in microglia. For this purpose, 8-OH-dG immunostaining was used to detect cellular ROS. CD11b and 8-OH-dG staining were increased in the prefrontal cortex (Figure 8, *P* < 0.05), however, minocycline administration attenuated CD11b expression and ROS overproduction in the microglia in the prefrontal cortex on day 14 after SNI (Figure 8, *P* < 0.05). There was no difference between the Sham group and the Sham + Mino group in the expression of CD11b and 8-OH-dG (Figure 8).

## Discussion

In the present study, we verified that SNI could induce the comorbidity of neuropathic pain and depression, M1 microglia polarization and amplified inflammatory cytokines as well as increased oxidative stress. Of note, we found that chronic minocycline administration reversed these abnormalities, probably mediated by inhibiting M1 microglia polarization and simultaneously promoting M2 microglia polarization. These findings may help to provide an avenue to treat neuropathic pain and a new explanation for the comorbidity of neuropathic pain and depression.

The comorbidity of chronic pain and depression has been well established in the literature (Bair et al., 2003; Miller and Cano, 2009). The common used models of comorbidity of neuropathic pain-induced depression include chronic constriction injury (Bennett and Xie, 1988), spinal nerve ligation (Kim and Chung, 1992), spinal nerve transection (Hu et al., 2010), and the SNI (Wang et al., 2011). In the present study, SNI-induced mechanical allodynia and depressive-like behaviors were confirmed by increased withdrawal threshold and immobility time and reduced sucrose preference, suggesting SNI induced the comorbidity of chronic pain and depression.

Previous studies have shown that the monoaminergic system, hypothalamic-pituitary-adrenal axis, neuroimmune responses, and neurotransmitters/neuromodulators are involved in the pathogenesis of the comorbidity of chronic pain and depression (Tsigos and Chrousos, 2002; Jasmin et al., 2003; Schatzberg, 2004; Andre et al., 2005; Gameiro et al., 2006; Walker et al., 2014), however the cellular mechanism underlying this comorbidity relationship remains largely to be elucidated. Accumulating evidence has suggested the key role of neuroinflammation mediated by microglia in linking neuropathic pain and depression (Burke et al., 2014; Fiore and Austin, 2016). It is reported that pro-inflammatory cytokines led to hyperalgesia (Schiavuzzo et al., 2015); meanwhile, pro-inflammatory cytokines also induced depressive-like behaviors in animals (Norman et al., 2010); In human studies, pro-inflammatory cytokines such as TNF-α and IL-1β are increased in the cerebrospinal fluid and blood in patients with chronic neuropathic pain (Alexander et al., 2005), suggesting a critical role of M1-mediated neuroinflammation in this disorder. Similar to peripheral macrophages, microglia are often classified into pro-inflammatory (M1) and alternatively activated (M2) phenotypes, in which the M1 phenotype was originally induced by lipopolysaccharide (LPS) or interferon (IFN)-γ stimulation, and the M2 phenotype by interleukin IL-4, IL-10, or IL-13 (Colton and Wilcock, 2010). It has been well documented that microglia M1/M2 polarization plays a pivotal role in controlling the balance between promotion and suppression of inflammation (Tao et al., 2016). In line with this notion, a recent study has shown that a transition to M1 phenotype is detrimental to neuroinflammation and neuropathic pain after SNI, whereas a failed M2 microglia response may contribute to the change from protective responses to harmful results because a failed inflammation control and decreased neuroprotective factors produced by microglia (Cherry et al., 2014). Thus, selective suppression of M1 and increase in the M2 polarization of microglia could be a potential strategy for treatment.

Minocycline, a commonly used inhibitor of microglia activation, has been proven to be neuroprotective in several animal models, such as experimental autoimmune encephalomyelitis (Howell et al., 2010), Parkinson's disease (Shan et al., 2011), and Alzheimer s disease (Malm et al., 2008). Minocycline also reduces the release of pro-inflammatory cytokines in the spinal cord and alleviates the neuropathic pain behaviors (Raghavendra et al., 2003b). In the present study, we studied the effects of minocycline on the neuropathic pain-induced depression and microglia M1/M2 markers in the prefrontal cortex and found that minocycline treatment attenuated the comorbidity by decreasing the expression of M1 microglia markers and associated pro-inflammatory cytokines and increasing the expression of the M2 microglia markers and associated anti-inflammatory cytokine. These results suggested minocycline modulated microglia polarization by boosting the M2 over the pro-inflammatory phenotype. In support with our results, it has been shown that minocycline has therapeutic effects on working memory deficit and depressive-like behaviors in these models (Hinwood et al., 2012; Majidi et al., 2016). However, another study showed that administration of minocycline attenuated the induction of M1 microglia markers but did not affect the transient enhancement of M2 microglia markers during the early pathogenesis phase (Kobayashi et al., 2013). A clear explanation for this discrepancy is still lacking because of our insufficient knowledge on the nature of microglia activation and the mechanisms of minocycline in controlling microglia in neuropathic pain models. However, our study did suggest that chronic minocycline administration differentially regulates the expression of M1/M2 microglia markers or relevant cytokines in the prefrontal cortex in an animal model of SNI.

There are some limitations in our study protocol. It should be mentioned that we only studied the prefrontal cortex here, which cannot represent the global brain expression of M1/M2 markers of microglia. However, we selected this brain region to study because one prior study has shown that the medial prefrontal cortex is an important region for the comorbidity of chronic pain and depression or anxiety (Burke et al., 2015). Consistently, the prefrontal cortex was found to involve in neuroimmune response in the olfactory bulbectomised model and following nerve injury (Apkarian et al., 2006; Myint et al., 2007; Borre et al., 2012), demonstrating the prefrontal cortex is implicated in the interaction between depression and pain. Second, it is important to measure the polarization markers at different time points, but we have detected only a time point. Longitudinal studies will be required in the future. Third, the negative results of serum IL-6 level may be the consequence of small sample sizes. Fourth, we cannot conclude that IL-4 is the primary M2-polarizing signal induced by chronic minocycline administration and a more extensive array analysis will be performed in the future to determine the hierarchy of M2 regulation.

In conclusion, our results suggested that SNI-induced neuropathic pain is associated with an enhanced pro-inflammatory M1/M2 microglia ratio. Notably, chronic minocycline administration alleviated the behavioral abnormities and selectively inhibited the microglia polarization to a pro-inflammatory state while attained an anti-inflammatory state after SNI. Our study provided additional evidence indicating the potential molecular mechanisms by which chronic minocycline administration can treat neuropathic pain and explained why patients with neuropathic pain frequently suffer from depression: because M1 polarization is a risk factor for depression. Therefore, future intervention should aim to prevent excessive microglia activities while retain their protective functions to treat the comorbidity of neuropathic pain and depression.

## Author contributions

NX, ZZ, XT, and MZ conceived and designed the experiments. NX, XT, and WP performed the experiment. WP, ZX, and GZ supervised acquisition of results and analyzed the data. NX, MJ, and JY wrote and refined the article.

## Funding

This study was supported by No. 81503053 from the National Natural Science Foundation of China.

### Conflict of interest statement

The authors declare that the research was conducted in the absence of any commercial or financial relationships that could be construed as a potential conflict of interest.
